# Influence of the *LILRA3* Deletion on Multiple Sclerosis Risk: Original Data and Meta-Analysis

**DOI:** 10.1371/journal.pone.0134414

**Published:** 2015-08-14

**Authors:** Miguel A. Ortiz, Concepción Núñez, David Ordóñez, José C. Alvarez-Cermeño, José E. Martínez-Rodriguez, Antonio J. Sánchez, Rafael Arroyo, Guillermo Izquierdo, Sunny Malhotra, Xavier Montalban, Antonio García-Merino, Elvira Munteis, Antonio Alcina, Manuel Comabella, Fuencisla Matesanz, Luisa M. Villar, Elena Urcelay

**Affiliations:** 1 Immunology Department, Hospital Clínico San Carlos, Instituto de Investigación Sanitaria San Carlos (IdISSC), Madrid, Spain; 2 Immunogenetics & Histocompatibility, Instituto de Investigación Sanitaria Puerta de Hierro, Majadahonda, Madrid, Spain; 3 Departments of Immunology and Neurology, Multiple Sclerosis Unit, Hospital Ramon y Cajal, (IRYCIS), Madrid, Spain; 4 Neurology, Hospital del Mar Medical Research Institute (IMIM), Barcelona, Spain; 5 Neuroimmunology, Hospital Universitario Puerta de Hierro, Majadahonda, Madrid, Spain; 6 Multiple Sclerosis Unit, Neurology Department. Hospital Clínico S. Carlos, Instituto de Investigación Sanitaria S. Carlos (IdISSC), Madrid, Spain; 7 Multiple Sclerosis Unit, Hospital Virgen Macarena, Sevilla, Spain; 8 Servei de Neurologia-Neuroimmunologia, Centre d'Esclerosi Múltiple de Catalunya (Cemcat), Institut de Recerca Vall d'Hebron (VHIR), Hospital Universitari Vall d'Hebron, Universitat Autònoma de Barcelona, Barcelona, Spain; 9 Department of Cell Biology and Immunology, Instituto de Parasitología y Biomedicina “López Neyra”, Consejo Superior de Investigaciones Científicas (IPBLN-CSIC), Granada, Spain; University of Oxford, UNITED KINGDOM

## Abstract

**Background:**

Multiple sclerosis (MS) is a neurodegenerative, autoimmune disease of the central nervous system. Genome-wide association studies (GWAS) have identified over hundred polymorphisms with modest individual effects in MS susceptibility and they have confirmed the main individual effect of the Major Histocompatibility Complex. Additional risk loci with immunologically relevant genes were found significantly overrepresented. Nonetheless, it is accepted that most of the genetic architecture underlying susceptibility to the disease remains to be defined. Candidate association studies of the leukocyte immunoglobulin-like receptor *LILRA3* gene in MS have been repeatedly reported with inconsistent results.

**Objectives:**

In an attempt to shed some light on these controversial findings, a combined analysis was performed including the previously published datasets and three newly genotyped cohorts. Both wild-type and deleted *LILRA3* alleles were discriminated in a single-tube PCR amplification and the resulting products were visualized by their different electrophoretic mobilities.

**Results and Conclusion:**

Overall, this meta-analysis involved 3200 MS patients and 3069 matched healthy controls and it did not evidence significant association of the *LILRA3* deletion [carriers of *LILRA3* deletion: p = 0.25, OR (95% CI) = 1.07 (0.95–1.19)], even after stratification by gender and the HLA-DRB1*15:*01* risk allele.

## Introduction

Multiple sclerosis (MS), a disease of the central nervous system involving chronic inflammation, axonal damage and demyelination, is likely caused by the interplay of genetic and environmental risk factors. The class II HLA-DRB1*15:*01* allele has been described as the main genetic susceptibility factor for the disease[1], although other immunological relevant genes have been also recently associated with MS predisposition [2].

Leukocyte immunoglobulin-like receptors (LILRs), also known as immunoglobulin-like transcripts (ILTs) or monocyte inhibitory receptors (MIRs), are broadly expressed on myeloid and lymphoid cells and play an important role in modulating innate and adaptive immune responses [3]. LILRA3 is a soluble protein of the LILR family that contains four Ig-like domains (D1, D2, D3 and D4) and acts as a soluble receptor for certain class I Major Histocompatibility Complex (MHC) antigens [4].

Members of the *LILR* gene family map to the leukocyte receptor complex on chromosome 19q13.4, a region that encodes at least 24 members of the immunoglobulin superfamily [5]. Reportedly, chromosome 19q13 is one of the genomic regions that exhibits clustering of susceptibility loci in several autoimmune diseases including MS [6, 7]. However, genome-wide association studies (GWAS) performed to date have not been able to detect any signal with significant association in this region when considering MS risk [8, 9]. GWAS benefit of a high throughput technology based on genotyping a growing number of single-nucleotide polymorphisms (SNPs), even over a million. Nonetheless, this is a limited strategy to detect any other kind of genetic variants such as deletions, unless they show high linkage disequilibrium with a SNP. An unprecedented advance unraveling the genetic architecture of complex diseases has been driven by these genome-wide studies, but a high percentage of the heritability of these disorders still remains unidentified [10].

The *LILRA3* gene exhibits a 6.7kb deletion comprising the first 6 of a total of 7 exons, resulting in a “null allele” because it removes all the regions coding Ig-like domains of the protein [11–13]. At present, little is known about its role in the immune system. The *LILRA3* gene has been associated with increased susceptibility to Sjögren's syndrome and to some subphenotypes of systemic lupus erythematosus [14, 15]. Increased levels of the soluble LILRA3 protein have been found in patients with rheumatoid arthritis [16]. Moreover, a study published by Du *et al*.[17] provided evidence for the functional *LILRA3* allele as a genetic risk factor only for male patients with rheumatoid arthritis. These data point to a potential genetic background shared by different autoimmune diseases, as described by some authors[18].

The association of the *LILRA3* deletion with MS susceptibility has been repeatedly studied [6, 19–21] yielding inconclusive results, some of them reporting significant effects while others being unable to confirm the association. Therefore, we aimed to perform a new analysis trying to ascertain the effect of the *LILRA3* deletion on MS risk in people with European ancestry. To do so, all previously published datasets and three newly genotyped cohorts were included in a meta-analysis. Further, we investigated the epistatic interaction between *LILRA3* and the classical MS risk factor HLA-DRB1*15:*01*, considering also the effect of gender previously described as a determinant in the *LILRA3* effect.

## Material and Methods

### Patients

A total of 1932 relapsing-remitting and secondary progressive (11% of total) MS patients of Spanish ancestry and 1599 ethnically matched controls was consecutively recruited from the following Spanish Hospitals: H. Clínico San Carlos and H. Ramón y Cajal, from Madrid, Central Spain (1422 patients and 841 controls); H. Virgen Macarena (Sevilla) and H. Virgen de las Nieves (Granada) from Andalusia, South of Spain (236 patients and 443 controls); and H. Vall d´Hebron (Catalonia), Northeast of Spain (274 patients and 315 controls). Both clinical forms are considered two stages of the same disease, to which we will refer here as relapse-onset MS (R-MS). Patients were diagnosed on the basis of McDonald criteria [22] and were recruited after written informed consent. The Ethics Committee of the Hospital Clínico San Carlos (CEIC- Comité Etico de Investigación Clínica) approved this study.

### Genotyping

The presence/ absence of the *LILRA3* deletion in our samples was studied as previously described [20]. Briefly, both wild-type and deleted *LILRA3* alleles were discriminated in a single-tube PCR amplification and the resulting products were visualized by their different electrophoretic mobility in regular agarose gels.

### Literature search and data abstraction

Genetic association studies investigating the role of *LILRA3* on MS susceptibility in European populations were identified by two independent researchers using the following terms: “LILRA3 or ILT6 or 19q13” and “multiple sclerosis” in NCBI's PubMed, Web of Science, Embase, Scopus and Google Scholar databases and by inspecting cross-references in related publications. All the articles published before June 2015 were included. No discrepancies between researchers were found. After review of 36 full-text articles, four of them published in peer-reviewed journals and including genotyping data from five populations were selected for meta-analysis, and the other 32 articles were excluded (see S1 Table, S1 Checklist and S1 PRISMA Checklist.). Raw data were requested to the corresponding author when not included in the study.

### Meta-analysis

**Fig 1:**
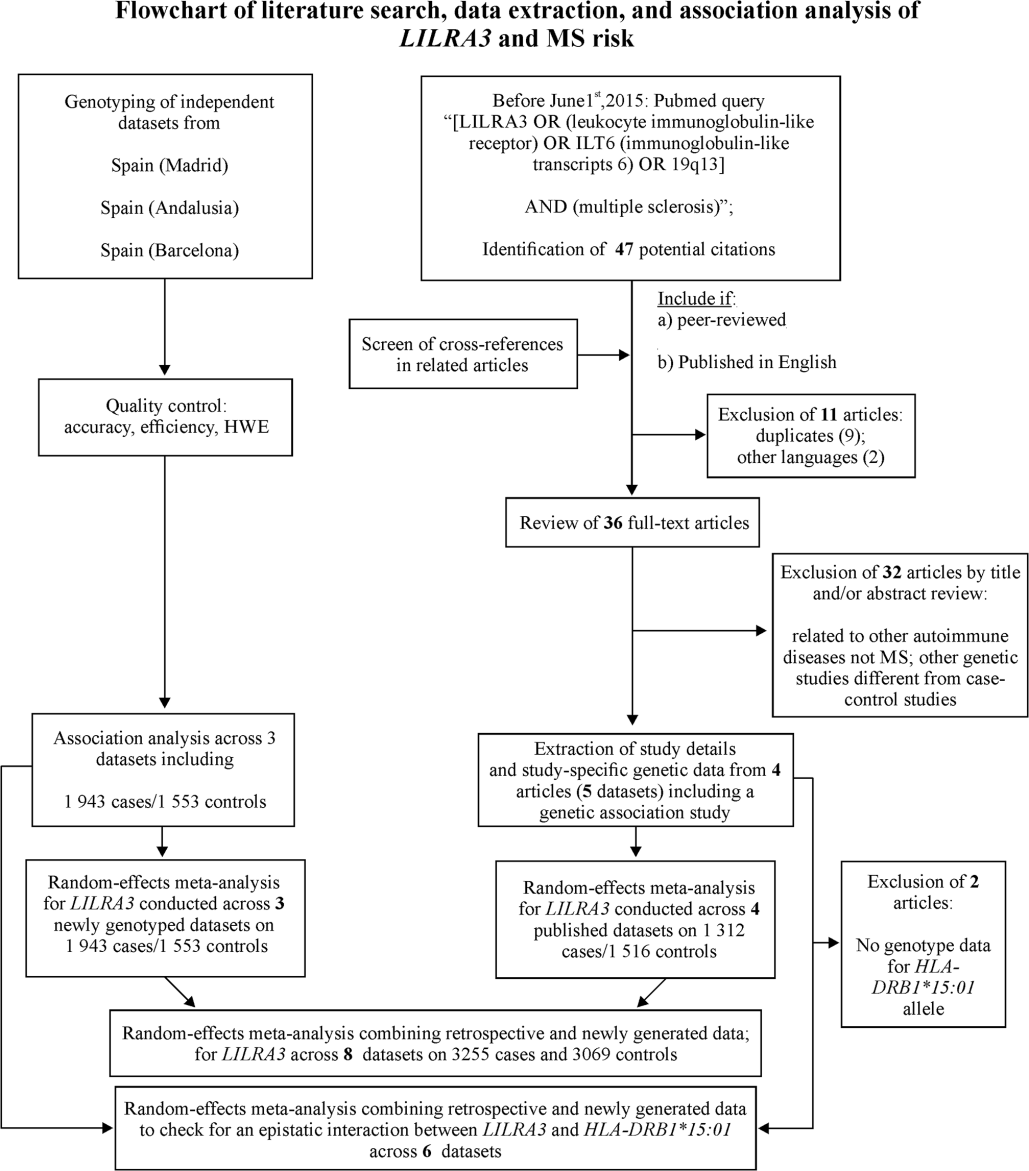
Flowchart of literature search, data extraction and association analysis of *LILRA3* and MS risk.

Meta-analyses were performed with the published and the newly generated datasets, based on the previously reported strategy followed by Lill *et al*. [23], Fig 1. The Cochran-Mantel-Haenszel method implemented in Review Manager (RevMan) version 5.0 (Copenhagen: The Nordic Cochrane Centre, The Cochrane Collaboration, 2008) was used to calculate combined odds ratios (OR) and 95% confidence intervals (CI). P-values <0.05 were considered statistically significant. The Der Simonian and Laird random effects model was used according to the results of the tests of heterogeneity. A sensitivity analysis was performed to test the relative influence of each study: studies were sequentially dropped, and the effect on the change in the overall degree of heterogeneity was quantified with the I^2^ statistic.

### Statistical analysis

Hardy-Weinberg proportions (HWE) were tested in controls. Statistical analyses were performed by using the SPSS 17.0 package (SPSS Inc, Chicago, IL). Allelic and genotypic frequency comparisons were analyzed using the χ^2^ test of a Fisher exact test (when expected values were below 5). Odds ratio (ORs) were calculated and their 95% CIs were estimated with the Cornfield method. Power calculations were performed by using the statistical software package Statcal Epi Info v.6.

## Results

### Association of the *LILRA3* deletion with R-MS susceptibility

**Fig 2:**
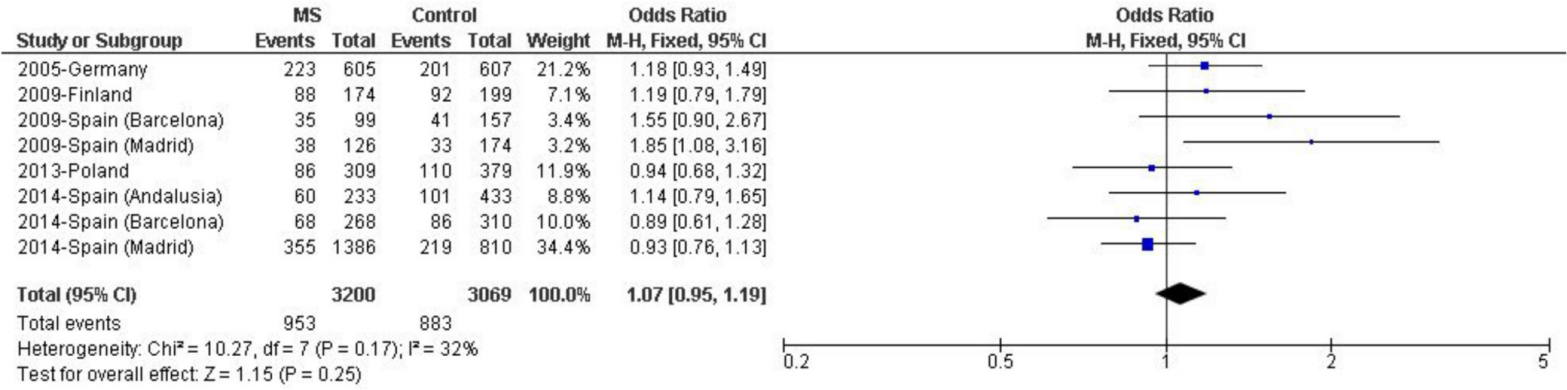
Meta-analysis assessing the association of *LILRA3* deletion with MS risk in populations of European descent. Forest plot of random effects with study-specific ORs (squares) and 95% CIs (lines) calculated for each individual dataset. Pooled OR (diamond) and 95% CI were calculated combining all datasets.

Four previously published reports have studied the association of the *LILRA3* deletion with MS susceptibility risk in five individual datasets (Fig 1). Meta-analysis across these published datasets yielded a statistically significant effect in carriers of the *LILRA3* deletion [p = 0.03; OR (95% CI) = 1.19 (1.02–1.40)], with modest evidence of heterogeneity (I^2^ = 25%). Subsequently, a combined analysis of the previously published datasets adding the data of the three newly genotyped independent cohorts was performed. Overall, this meta-analysis including 3200 MS patients and 3069 matched healthy controls did not reveal significant evidence for association and yielded an effect size close to one [Fig 2, carriers of *LILRA3* deletion: p = 0.25, OR (95% CI) = 1.07 (0.95–1.19)] with moderate evidence for heterogeneity (I^2^ = 32%). The number of individuals included would allow the detection of an effect with OR> 1.17, with a statistical power over 80%. A further sensitivity analysis was performed in this study, which provided I^2^ values ranging from 2% to 41%: the analysis excluding the dataset “2009-Spain (Madrid)” led to the lowest heterogeneity, and again statistical significance was not observed (p = 0.49).

### Analysis of the epistatic interaction between the *LILRA3* deletion and the HLA-DRB1*15:*01* allele

**Fig 3:**
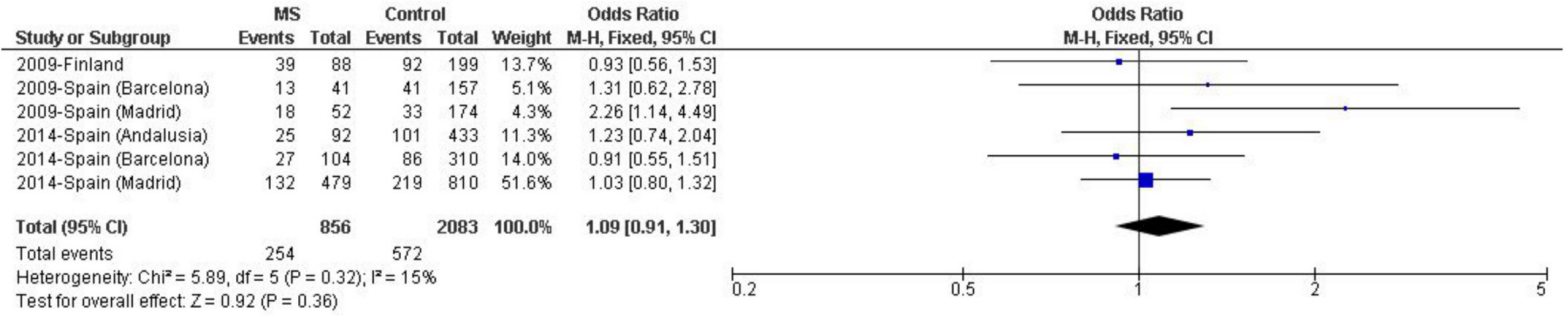
Random effects meta-analysis including association studies of the *LILRA3* deletion with MS risk in HLA-DRB1*15:*01* carriers from populations of European descent. Study-specific ORs (squares) and 95% CIs (lines) were calculated for each individual dataset. Pooled OR and 95% CI were calculated combining all datasets. Del+/15:01+: carrier of *LILRA3* deletion and HLA-DRB1*15:*01* allele.

To check for an epistatic interaction between the *LILRA3* deletion and the classical MS risk factor HLA-DRB1*15:*01*, a meta-analysis of those datasets including both variants was performed (Fig 3). The meta-analysis did not evidence any significant epistatic interaction when these two genetic factors were considered [p = 0.36, OR (95% CI) = 1.09 (0.91–1.30)]. Conversely, to ascertain whether a possible effect of the *LILRA3* deletion might be restricted to subjects lacking the HLA-DRB1*15:*01* allele, the same analysis was performed in this group of patients, but a significant difference was not observed [p = 0.74, OR (95% CI) = 1.03 (0.88–1.20)].

### Analysis of the epistatic interaction between the *LILRA3* deletion and HLA-DRB1*15:*01* allele after gender-stratification

**Fig 4:**
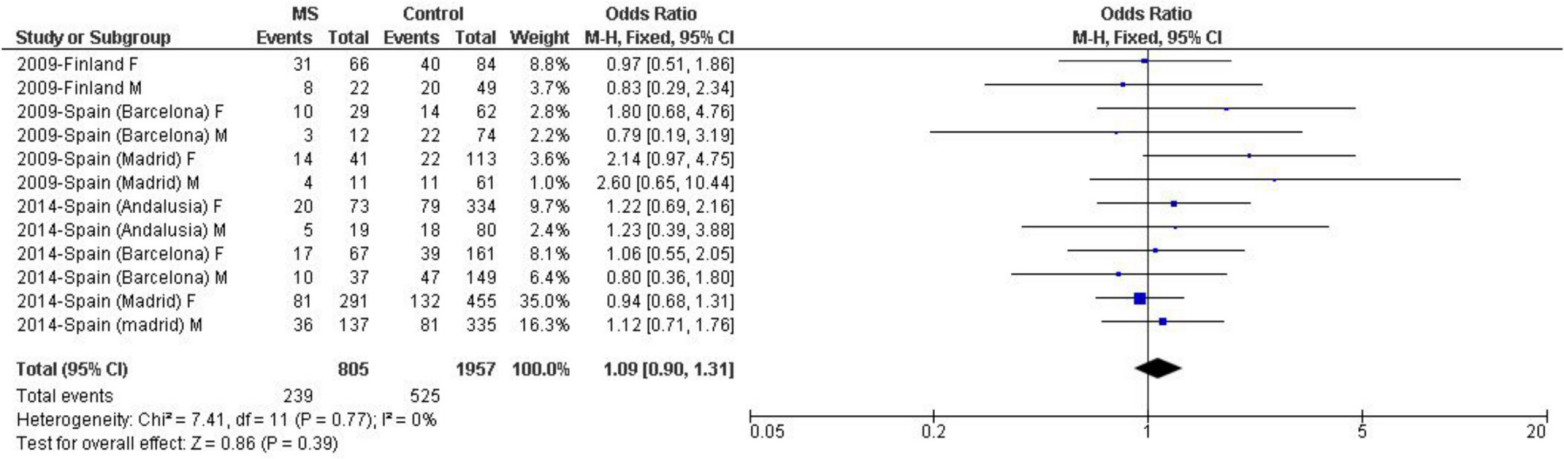
Random effects meta-analysis of the association studies of the *LILRA3* deletion with MS risk in HLA-DRB1*15:*01* carriers from populations of European descent stratified by gender. Study-specific ORs (squares) and 95% CIs (lines) were calculated for each individual dataset. Pooled OR and 95% CI were calculated combining all datasets. Del+/15:01+: carrier of both *LILRA3* gene deletion and HLA-DRB1*15:*01* allele, M: male, F: female.

Further analysis was performed stratifying by gender the *LILRA3* deletion/ HLA-DRB1*15:*01* double-positive subjects and again, a significant difference was not detected [Fig 4, p = 0.39, OR (95% CI) = 1.09 (0.90–1.31)] with total homogeneity of strata (I^2^ = 0%). In addition, separated analyses in male and female groups were also performed, with similar results observed in both cases [male: p = 0.76, OR (95% CI) = 1.05 (0.76–1.47) and female: p = 0.40, OR (95% CI) = 1.10 (0.88–1.38)]. Neither significant evidence of sex-bias was observed in carriers of the *LILRA3* deletion lacking HLA-DRB1*15:*01* (data not shown).

## Discussion

The role of genetics in MS susceptibility is highly accepted and broadly described in the literature. At present, the greatest advances in the study of this genetic component have been achieved through GWAS, which analyze thousands of patients and ethnically matched controls searching for evidences to explain MS pathogenesis. However, this kind of studies does not explore genetic variants other than SNPs and the heritability underlying this and other complex diseases persists partially unexplained.

Previously reported studies tried to evaluate the influence of the *LILRA3* deletion on MS risk; however, their results were not fully concordant and it was difficult to draw a firm conclusion regarding the impact of this gene on MS. Two studies performed in German [21] and Spanish populations [20] showed a significant association of the *LILRA3* deletion with R-MS, with the strongest association found in the Spanish study. A MS cohort of French origin showed a high *LILRA3-*deletion frequency (46.1%), but the study included no matched French healthy controls[21], and it has not been included in our meta-analysis. Conversely, two studies performed in Finnish [6] and Polish populations [19] could not replicate the association. Therefore, a risk of publication bias does not seem to be present in the study of this gene. The meta-analysis combining the mentioned cohorts evidenced a statistically significant association of *LILRA3* deletion with MS risk [p = 0.03, OR (95% CI) = 1.19 (1.02–1.40)], with modest evidence of heterogeneity (I^2^ = 25%).

Considering that small sample sizes might explain the inconsistent results observed in the different reports, we aimed to ascertain the influence of the *LILRA3* deletion on R-MS susceptibility by studying this association in a large number of individuals from three different Spanish locations (Barcelona, Madrid and Andalusia) and performing a meta-analysis with the available data. Overall, our study with 6380 individuals (3257 MS patients and 3123 healthy matched controls) did not reveal any significant difference when the distribution of the *LILRA3* deletion in MS patients and controls was compared [p = 0.25, OR (95% CI) = 1.07 (0.95–1.19)], albeit in the context of a moderate evidence of heterogeneity (I^2^ = 32%). Substantial attenuation of the latter was achieved after the sensitivity analysis, which showed that the exclusion of the “2009-Spain (Madrid)” cohort reported by Ordonez *et al*. [20] led to a lower heterogeneity in the overall meta-analysis, still lacking significant association (p = 0.49). Interestingly, the maximum reduction of heterogeneity was reached when the study with the highest impact on MS risk was removed from the meta-analysis, raising the possibility that the effect once observed was driven by a strong signal in this cohort. In conclusion, it seems that the *LILRA3* deletion by itself does not alter MS susceptibility.

Additionally, we also considered two other important factors: HLA-DRB1*15:*01* as the main risk allele described in MS and the putative gender bias,which might underlie the inconsistent results reported in the different studies. As previously described by Ordonez *et al*. [20],we assessed whether there was a synergistic effect between HLA-DRB1*15:*01* and the *LILRA3* deletion in order to confer MS risk, but no significant epistatic interaction was observed (p = 0.36). Moreover, to evaluate whether the *LILRA3* deletion presented a possible gender bias in MS risk, as described by Du *et al*. in rheumatoid arthritis susceptibility [17], the epistatic interaction was also studied after gender stratification. Again, no significant differences were observed in male or female MS patients (p = 0.76 and p = 0.40, respectively), although two of the initial datasets were excluded as they lacked both gender- and DRB1*15:*01*- detailed information. Therefore, stratifications considering either the HLA-DRB1*15:*01* allele by itself or including also gender information did not evidence global association of *LILRA3* deletion with MS risk, demonstrating that a more stringent stratification of patients did not explain previous results.

Some works seemed to support the role of the *LILRA3* gene conferring risk to different autoimmune diseases and this gene would be another example of the well-established concept of shared genetic background among autoimmune diseases [14, 15, 17]. However, some of those studies warrant validation in independent cohorts or populations, provided that the studies including the greatest number of subjects have been performed in Asian populations. The frequency of the 6.7kb deletion differ greatly among ethnic groups, with extremely high values in Asians (0.56–0.84), and much lower values in Europeans (0.17) or Africans (0.10) [24]. It is well known that Asian and Caucasian populations show specific risk variants given the different genetic drift and the resulting different patterns of linkage disequilibrium (LD). In Asian populations, one SNP (rs103294) was found in LD (r^2^ = 0.83) with the *LILRA3* deletion and, therefore, it was selected by Du *et al*. to study the association of the *LILRA3* gene with rheumatoid arthritis susceptibility[17]. In Caucasian populations, the LD pattern between the aforementioned SNP and the *LILRA3* deletion has not been formally reported. Nonetheless, this SNP was included in an analysis of immune-related loci that identified 48 new risk variants for MS [2]. Although the allelic frequencies of rs103294 and other polymorphisms close to the *LILRA3* gene are similar to that displayed by the *LILRA3*deletion, no association with either MS or rheumatoid arthritis, psoriasis, type 1 diabetes or celiac disease risk was identified in this region, in agreement with the present results.

Our results are also concordant with those found in psoriasis, that reported lack of significant association between the *LILRA3* deletion and the disease in a European population [25], although this finding warrants replication as well. All these lines of evidence together indicated that *LILRA3* does not seem to be underlying MS pathogenesis. Understanding the full load of genetic factors contributing to disease risk has potential for profound improvements in health care. Yet, the known genetic factors account for only a small portion of the estimated heritability of complex phenotypes and every effort to overcome this impasse should be emphasized.

## Supporting Information

S1 Checklist Checklist of meta-analysis. (DOCX)

S1 PRISMA Checklist PRISMA checklist. (DOC)

S1 Table List of 32 full-articles excluded from the meta-analysis due to lack of pertinent data. (DOCX)
