# Supplementary material for: Influence of the LILRA3 Deletion on Multiple Sclerosis Risk: Original Data and Meta-Analysis
Source: PLoS One. 2015 Aug 14;10(8):e0134414. doi: 10.1371/journal.pone.0134414 (PMC4537248; doi:10.1371/journal.pone.0134414)
Supplement: S1 Table — (DOCX) [file pone.0134414.s003.docx]

**Supplementary file 2**

1. Du, Y., Cui, Y., Liu, X., Hu, F., Yang, Y., Wu, X., Ma, X., Zuo, X., Sheng, Y., Xu, J., Zhu, P., Sun, L., Hong, N., Zhang, X., Guo, J., and Li, Z. 2014. Contribution of functional LILRA3, but not nonfunctional LILRA3, to sex bias in susceptibility and severity of anti-citrullinated protein antibody-positive rheumatoid arthritis. Arthritis Rheumatol **66:** 822.

2. An, H., Chandra, V., Piraino, B., Borges, L., Geczy, C., McNeil, H. P., Bryant, K., and Tedla, N. 2010. Soluble LILRA3, a potential natural antiinflammatory protein, is increased in patients with rheumatoid arthritis and is tightly regulated by interleukin 10, tumor necrosis factor-alpha, and interferon-gamma. J Rheumatol **37:** 1596.

3. Kabalak, G., Dobberstein, S. B., Matthias, T., Reuter, S., The, Y. H., Dorner, T., Schmidt, R. E., and Witte, T. 2009. Association of immunoglobulin-like transcript 6 deficiency with Sjogren's syndrome. Arthritis Rheum **60:** 2923.

4. Hirayasu, K., Ohashi, J., Kashiwase, K., Takanashi, M., Satake, M., Tokunaga, K., and Yabe, T. 2006. Long-term persistence of both functional and non-functional alleles at the leukocyte immunoglobulin-like receptor A3 (LILRA3) locus suggests balancing selection. Hum Genet **119:** 436.

5. Kabalak, G., Koch, S., Dobberstein, B., The, Y. H., Matthias, T., Schnarr, S., Schmidt, R. E., and Witte, T. 2007. Immunoglobulin-like transcripts as risk genes for autoimmunity. Ann N Y Acad Sci **1110:** 10.

6. Sulonen, A. M., Kallio, S. P., Ellonen, P., Suvela, M., Elovaara, I., Koivisto, K., Pirttila, T., Reunanen, M., Tienari, P. J., Palotie, A., Peltonen, L., and Saarela, J. 2009. No evidence for shared etiology in two demyelinative disorders, MS and PLOSL. J Neuroimmunol **206:** 86.

7. Datta, P., Harbo, H. F., Ryder, L. P., Akesson, E., Benedikz, J., Celius, E. G., Andersen, O., Myhr, K. M., Sandberg-Wollheim, M., Hillert, J., Svejgaard, A., Sorensen, P. S., Spurkland, A., and Oturai, A. 2007. A follow-up study of Nordic multiple sclerosis candidate gene regions. Mult Scler **13:** 584.

8. Haghighi, S., Andersen, O., Nilsson, S., Rydberg, L., and Wahlstrom, J. 2006. A linkage study in two families with multiple sclerosis and healthy members with oligoclonal CSF immunopathy. Mult Scler **12:** 723.

9. Rosche, B., Cepok, S., Stei, S., Vogel, F., Grummel, V., Hoffmann, S., Kroner, A., Maurer, M., Rieckmann, P., Sommer, N., and Hemmer, B. 2004. The role of the polio virus receptor and the herpesvirus entry mediator B genes for the development of MS. J Neuroimmunol **156:** 171.

10. Johansson, C. M., Zunec, R., Garcia, M. A., Scherbarth, H. R., Tate, G. A., Paira, S., Navarro, S. M., Perandones, C. E., Gamron, S., Alvarellos, A., Graf, C. E., Manni, J., Berbotto, G. A., Palatnik, S. A., Catoggio, L. J., Battagliotti, C. G., Sebastiani, G. D., Migliaresi, S., Galeazzi, M., Pons-Estel, B. A., and Alarcon-Riquelme, M. E. 2004. Chromosome 17p12-q11 harbors susceptibility loci for systemic lupus erythematosus. Hum Genet **115:** 230.

11. Pericak-Vance, M. A., Rimmler, J. B., Haines, J. L., Garcia, M. E., Oksenberg, J. R., Barcellos, L. F., Lincoln, R., Hauser, S. L., Cournu-Rebeix, I., Azoulay-Cayla, A., Lyon-Caen, O., Fontaine, B., Duhamel, E., Coppin, H., Brassat, D., Roth, M. P., Clanet, M., Alizadeh, M., Yaouanq, J., Quelvennec, E., Semana, G., Edan, G., Babron, M. C., Genin, E., and Clerget-Darpoux, F. 2004. Investigation of seven proposed regions of linkage in multiple sclerosis: an American and French collaborative study. Neurogenetics **5:** 45.

12. Jonasdottir, A., Thorlacius, T., Fossdal, R., Jonasdottir, A., Benediktsson, K., Benedikz, J., Jonsson, H. H., Sainz, J., Einarsdottir, H., Sigurdardottir, S., Kristjansdottir, G., Sawcer, S., Compston, A., Stefansson, K., and Gulcher, J. 2003. A whole genome association study in Icelandic multiple sclerosis patients with 4804 markers. J Neuroimmunol **143:** 88.

13. Ban, M., Sawcer, S. J., Heard, R. N., Bennetts, B. H., Adams, S., Booth, D., Perich, V., Setakis, E., Compston, A., and Stewart, G. J. 2003. A genome-wide screen for linkage disequilibrium in Australian HLA-DRB1*1501 positive multiple sclerosis patients. J Neuroimmunol **143:** 60.

14. Haines, J. L., Bradford, Y., Garcia, M. E., Reed, A. D., Neumeister, E., Pericak-Vance, M. A., Rimmler, J. B., Menold, M. M., Martin, E. R., Oksenberg, J. R., Barcellos, L. F., Lincoln, R., and Hauser, S. L. 2002. Multiple susceptibility loci for multiple sclerosis. Hum Mol Genet **11:** 2251.

15. Lucotte, G. L. 2002. Confirmation of a gene for multiple sclerosis (MS) to chromosome region 19q13.3. Genet Couns **13:** 133.

16. D'Alfonso, S., Mellai, M., Giordano, M., Pastore, A., Malferrari, G., Naldi, P., Repice, A., Liguori, M., Cannoni, S., Milanese, C., Caputo, D., Savettieri, G., and Momigliano-Richiardi, P. 2002. Identification of single nucleotide variations in the coding and regulatory regions of the myelin-associated glycoprotein gene and study of their association with multiple sclerosis. J Neuroimmunol **126:** 196.

17. Reunanen, K., Finnila, S., Laaksonen, M., Sumelahti, M. L., Wikstrom, J., Pastinen, T., Kuokkanen, S., Saarela, J., Uimari, P., Ruutiainen, J., Ilonen, J., Peltonen, L., and Tienari, P. J. 2002. Chromosome 19q13 and multiple sclerosis susceptibility in Finland: a linkage and two-stage association study. J Neuroimmunol **126:** 134.

18. Masterman, T., Zhang, Z., Hellgren, D., Salter, H., Anvret, M., Lilius, L., Lannfelt, L., and Hillert, J. 2002. APOE genotypes and disease severity in multiple sclerosis. Mult Scler **8:** 98.

19. Schmidt, S., Barcellos, L. F., DeSombre, K., Rimmler, J. B., Lincoln, R. R., Bucher, P., Saunders, A. M., Lai, E., Martin, E. R., Vance, J. M., Oksenberg, J. R., Hauser, S. L., Pericak-Vance, M. A., and Haines, J. L. 2002. Association of polymorphisms in the apolipoprotein E region with susceptibility to and progression of multiple sclerosis. Am J Hum Genet **70:** 708.

20. Pericak-Vance, M. A., Rimmler, J. B., Martin, E. R., Haines, J. L., Garcia, M. E., Oksenberg, J. R., Barcellos, L. F., Lincoln, R., Goodkin, D. E., and Hauser, S. L. 2001. Linkage and association analysis of chromosome 19q13 in multiple sclerosis. Neurogenetics **3:** 195.

21. Weinshenker, B. G., Hebrink, D., Kantarci, O. H., Schaefer-Klein, J., Atkinson, E., Schaid, D., and McMurray, C. M. 2001. Genetic variation in the transforming growth factor beta1 gene in multiple sclerosis. J Neuroimmunol **120:** 138.

22. Xu, C., Dai, Y., Lorentzen, J. C., Dahlman, I., Olsson, T., and Hillert, J. 2001. Linkage analysis in multiple sclerosis of chromosomal regions syntenic to experimental autoimmune disease loci. Eur J Hum Genet **9:** 458.

23. Green, A. J., Barcellos, L. F., Rimmler, J. B., Garcia, M. E., Caillier, S., Lincoln, R. R., Bucher, P., Pericak-Vance, M. A., Haines, J. L., Hauser, S. L., and Oksenberg, J. R. 2001. Sequence variation in the transforming growth factor-beta1 (TGFB1) gene and multiple sclerosis susceptibility. J Neuroimmunol **116:** 116.

24. D'Alfonso, S., Nistico, L., Bocchio, D., Bomprezzi, R., Marrosu, M. G., Murru, M. R., Lai, M., Massacesi, L., Ballerini, C., Repice, A., Salvetti, M., Montesperelli, C., Ristori, G., Trojano, M., Liguori, M., Gambi, D., Quattrone, A., Tosi, R., and Momigliano-Richiardi, P. 2000. An attempt of identifying MS-associated loci as a follow-up of a genomic linkage study in the Italian population. J Neurovirol **6 Suppl 2:** S18.

25. Barcellos, L. F., Thomson, G., Carrington, M., Schafer, J., Begovich, A. B., Lin, P., Xu, X. H., Min, B. Q., Marti, D., and Klitz, W. 1997. Chromosome 19 single-locus and multilocus haplotype associations with multiple sclerosis. Evidence of a new susceptibility locus in Caucasian and Chinese patients. Jama **278:** 1256.

26. Gustavsen, M. W., Viken, M. K., Celius, E. G., Berge, T., Mero, I. L., Berg-Hansen, P., Aarseth, J. H., Myhr, K. M., Sondergaard, H. B., Sellebjerg, F., Oturai, A. B., Hillert, J., Alfredsson, L., Olsson, T., Kockum, I., Lie, B. A., and Harbo, H. F. 2014. Oligoclonal band phenotypes in MS differ in their HLA class II association, while specific KIR ligands at HLA class I show association to MS in general. J Neuroimmunol **274:** 174.

27. Du, Y., Cui, Y., Liu, X., Hu, F., Yang, Y., Wu, X., Liu, X., Ma, X., Zuo, X., Sheng, Y., Liu, X., Xu, J., Zhu, P., Sun, L., Hong, N., Zhang, X., Guo, J., and Li, Z. 2014. Contribution of functional LILRA3, but not nonfunctional LILRA3, to sex bias in susceptibility and severity of anti-citrullinated protein antibody-positive rheumatoid arthritis. Arthritis Rheumatol **66:** 822.

28. Garcia-Leon, J. A., Lopez-Gomez, C., Orpez-Zafra, T., Reyes-Garrido, V., Marin-Banasco, C., Oliver-Martos, B., Fernandez, O., and Leyva, L. 2014. Killer-cell immunoglobulin-like receptor expression on lymphocyte subsets in multiple sclerosis patients treated with interferon-beta: evaluation as biomarkers for clinical response. CNS Drugs **28:** 559.

29. Rizzo, R., Gentili, V., Casetta, I., Caselli, E., De Gennaro, R., Granieri, E., Cassai, E., Di Luca, D., and Rotola, A. 2012. Altered natural killer cells' response to herpes virus infection in multiple sclerosis involves KIR2DL2 expression. J Neuroimmunol **251:** 55.

30. Garcia-Leon, J. A., Pinto-Medel, M. J., Garcia-Trujillo, L., Lopez-Gomez, C., Oliver-Martos, B., Prat-Arrojo, I., Marin-Banasco, C., Suardiaz-Garcia, M., Maldonado-Sanchez, R., Fernandez-Fernandez, O., and Leyva-Fernandez, L. 2011. Killer cell immunoglobulin-like receptor genes in Spanish multiple sclerosis patients. Mol Immunol **48:** 1896.

31. Lorentzen, A. R., Karlsen, T. H., Olsson, M., Smestad, C., Mero, I. L., Woldseth, B., Sun, J. Y., Senitzer, D., Celius, E. G., Thorsby, E., Spurkland, A., Lie, B. A., and Harbo, H. F. 2009. Killer immunoglobulin-like receptor ligand HLA-Bw4 protects against multiple sclerosis. Ann Neurol **65:** 658.

32. Jelcic, I., Hsu, K. C., Kakalacheva, K., Breiden, P., Dupont, B., Uhrberg, M., Martin, R., Munz, C., and Lunemann, J. D. 2012. Killer immunoglobulin-like receptor locus polymorphisms in multiple sclerosis. Mult Scler **18:** 951.

33. Aries PM, Witte T, Lamprecht P. [Report on the 34th meeting of the German Clinical Immunology Workgroup, Frankfurt, 03.-04.11.2006]. Z Rheumatol. 2007 Feb;66(1):63-4.

34. Molina-Monasterios MC, Molina-Abecia H. [Nasu Hakola disease: a report of the first two cases in Bolivia]. Rev Neurol. 2003 May 1-15;36(9):837-40.
